# *Blood Pressure Sunday:* Introducing Genomics to the Community Through Family History

**Published:** 2005-03-15

**Authors:** Debra Duquette, Velma Theisen, Rosalyn Beene-Harris, Janice Bach, Sharon Kardia, Catharine Wang

**Affiliations:** Michigan Department of Community Health; Michigan Department of Community Health, Lansing, Mich; Michigan Department of Community Health, Lansing, Mich; Michigan Department of Community Health, Lansing, Mich; University of Michigan–Center for Genomics and Public Health, Department of Epidemiology, Ann Arbor, Mich; University of Michigan–Center for Genomics and Public Health, Department of Health Behavior and Health Education, Ann Arbor, Mich

## Abstract

**Background:**

Family history of a chronic disease, such as high blood pressure, is an important predictor of future disease. The integration of genomics information into public health activities offers the opportunity to help raise awareness among populations at high risk for high blood pressure.

**Context:**

The prevalence of high blood pressure in blacks at any age is about twice that of whites. Detroit is second among major U.S. cities in the percentage of residents who are black (81.6%). According to data from the Behavioral Risk Factor Surveillance System 1998–2002, the perceived health status of Detroit respondents was one of the worst in Michigan; 17.4% of Detroit respondents reported no health care coverage; 69.6% reported being obese or overweight; and 33.1% reported no physical activity.

**Methods:**

The Michigan Department of Community Health and the University of Michigan's Center for Genomics and Public Health collaborated on a pilot program to develop a worksheet emphasizing the importance of personal family history of high blood pressure. The handout was distributed to individuals at primarily black, Detroit-area churches during an annual screening event for high blood pressure and stroke.

**Consequences:**

Approximately 500 handouts were distributed; a collaborative effort was achieved; genomics information was integrated into an existing program; the ability to reach churches in a predominantly black community was demonstrated; consumers reported interest in the subject matter; and an appropriate literacy level for the handout was attained.

**Interpretation:**

The strengths of this pilot program and suggested modifications may serve to guide others in genomics and/or chronic disease programs in future endeavors.

## Background

"It is not a question of *if*, but *when* and *how* the advances resulting from the Human Genome Project will be integrated into society, medicine and public health" (1). To integrate genomics into state public health activities, the Coordinating Center for Health Promotion within the Centers for Disease Control and Prevention (CDC) awarded Michigan and three other states a five-year cooperative agreement. The Michigan Department of Community Health's (MDCH's) genomics program is multidisciplinary, relying on internal partnerships with multiple chronic disease programs, including cardiovascular health. MDCH also relies on academic partnerships with the University of Michigan–Center for Genomics and Public Health (UM–CGPH) and two other academic centers. The MDCH Cardiovascular Health and Genomics Programs and UM–CGPH collaborated to translate genomic information about the importance of family history with the goal of raising awareness in a community at high risk for high blood pressure.

### High blood pressure

High blood pressure is a common and serious public health problem that affects about 30% of the U.S. adult population (2). High blood pressure, especially if untreated, can result in mortality and morbidity because of the complications of end-stage renal disease, coronary artery disease, and/or stroke. Risk factors for high blood pressure include:

- a family history of high blood pressure
- African American ancestry
- age 65 years or older
- low socioeconomic status
- overweight or obesity
- a sedentary lifestyle
- excess intake of dietary sodium and/or insufficient intake of potassium
- excess consumption of alcohol (3,4).

Although high blood pressure is serious, the majority of individuals diagnosed do not have their blood pressure controlled (3). Many factors contribute to poor control of blood pressure, such as lack of knowledge of the potential consequences, noncompliance, cost of managing a lifelong condition, and complexity of disease management. Prevention and control of a chronic disease begins with a good understanding of individual risk. 

High blood pressure can be prevented by a complementary application of strategies that targets the general population and individuals at high risk. Current national guidelines recommend nonpharmacologic therapy, including lifestyle modifications, for primary prevention and treatment of high blood pressure (5).

### Populations at risk for high blood pressure: blacks and the Detroit community

The prevalence of high blood pressure varies among populations. The most systematic comparison of ethnic groups has been between black and white individuals with hypertension (6). The prevalence of high blood pressure in blacks at any age is about twice that of whites (3,6). Blacks have been reported as having an earlier onset and greater frequency of stroke and renal failure, but a lower risk of coronary artery disease than whites (6). Sodium intake appears to be especially important in the etiology of high blood pressure for blacks (6). Genetic factors may influence the response to antihypertensive drugs; blacks are known to respond better to treatment with diuretics than with beta-blockers (6).

The Michigan Behavioral Risk Factor Surveillance System (BRFSS), combining 1997, 1999, and 2001 results, found that the proportion of blacks in Detroit who had ever been told by a health professional that they had high blood pressure was 34.9%, and the proportion of whites was 21.4% (7). The reported proportion of blacks with high blood pressure in Detroit is higher than that reported in Michigan adults (27.1%) and in the national median (25.6%) (7).

### Awareness of family history

A family health history reflects the outcome of numerous influences, including genetics, ethnicity, culture, and environment. The family health history holds important clues to current and future health risks for almost all chronic diseases, including high blood pressure. According to Yoon et al, "Evidence suggests that family history is useful for predicting disease when there are multiple family members affected, relationship among relatives is close and disease has an earlier onset than expected" (8).

An annual national mail survey, *HealthStyles,* recently included questions about the awareness of family history as a risk factor for disease. (*HealthStyles* is a proprietary database product of social marketing and public relations firm Porter Novelli, licensed by the CDC for audience analysis in health communication planning.) The survey indicated that 96.3% of respondents (n = 4345) considered knowledge of family history important, but only 29.8% were actively collecting the information (9). National efforts have begun to promote the collection and use of family history (9).

It has recently been stated that "certain subgroups of the population might benefit from targeted programs to raise awareness about the collection and recording of family health histories" (9). Information regarding family history of chronic diseases is also inexpensive to incorporate into already existing programs to increase awareness, educate, and encourage healthy behaviors and preventive measures.

### Genetics of high blood pressure

Family history has been recognized as a significant risk factor for high blood pressure since the 1930s and confirmed in numerous subsequent observational studies (6). However, the specific inheritance pattern of high blood pressure remains unknown. Most observations support multifactorial inheritance, with blood pressure having a continuous distribution influenced by multiple genes and environmental factors. As expected with multifactorial inheritance, correlation of blood pressure is seen with increasing biologic relatedness (6). Based on family studies, a history of high blood pressure in a first-degree relative increases the likelihood by about twofold that an individual will have high blood pressure (6).

In many families, high blood pressure is most likely a polygenic condition, meaning that multiple genes contribute to the development of high blood pressure. In other families, high blood pressure may be caused by a single gene that strongly influences blood pressure. Susceptibility genes have been localized, and candidate genes include those encoding angiotensinogen, angiotensin receptor-1, the beta-3 subunit of guanine nucleotide-binding protein, and tumor necrosis factor receptor-2 (10,11). In fact, routine DNA-based testing to predict common diseases may not occur for years (8). Yoon et al state, "In the meantime, family medical history represents a 'genomic tool' that can capture genetic susceptibility, shared environment and common behaviors in relation to disease risk" (12).

Family history is a tool that incorporates the genetic risk of an individual and can easily be used in public health practice. Thus, the MDCH and the UM–CGPH collaborated to develop a handout that emphasizes the importance of a personal family history of high blood pressure. The target audience was black adults in Detroit. The goal of the pilot project was to enable consumers to collect individual family history information and then to use this information to identify personal risks and possible health measures to prevent high blood pressure.

## Context

The city of Detroit is located in Wayne County, which is in southeast Michigan. It is the tenth largest city in the United States by population and is second among major U.S. cities in the percentage of blacks (81.6%) (Figure) (13). The median age among blacks living in Detroit is 40.4 years (14). Residents of Detroit are primarily from a low-wage workforce. As of 2000, Detroit ranked 88^th^ in household income out of the 100 largest U.S. cities (13).

**Figure:**
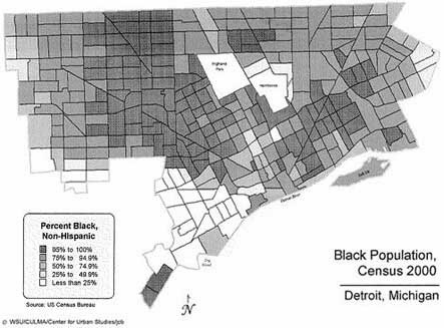
Black population of Detroit, Mich. Reproduced with permission from Wayne State University, Center for Urban Studies.

According to 1998–2002 BRFSS data, the perceived health status of Detroit respondents was one of the worst in Michigan (14). Among southeast Michigan respondents, Detroit had the highest proportion (17.4%) of respondents who reported no health care coverage (14). Additionally, 69.6% of Detroit respondents reported being obese or overweight, and 33.1% reported no physical activity in their leisure time, which was the highest in Michigan (14). In contrast, Detroit had one of the lowest rates of heavy alcohol use (3.9%) in southeast Michigan (14). As reflected in this BRFSS data, behavioral and environmental risk factors in the Detroit community may contribute to the larger proportion of individuals with hypertension (Table).

To test the feasibility of introducing the importance of family history and high blood pressure in a black population, we collaborated with an established project that offers stroke and blood pressure screening and education to faith-based groups in the Detroit area. The event, *Blood Pressure Sunday*, is a collaborative effort with MDCH and the American Heart Association, Greater Midwest Affiliate, and has been offered every May for five years as part of National Stroke Awareness and High Blood Pressure Month. During this month-long program, churches offer educational material, educational sessions, and screening after church services.

Participating churches identify a contact person, typically a parish nurse or health coordinator. Each contact person receives a workbook, material to distribute during the program, blood pressure measurement equipment, and one-day training by MDCH Cardiovascular Program staff. In the past, there have been up to 90 churches participating, 1907 individuals screened, and 270 individuals trained during the month-long event. The population reached is more than 75% black. A key component of the program is the emphasis on standardized, accurate blood pressure screening.

Based on the demographics of Detroit, *Blood Pressure Sunday* appeared to be an ideal program for targeted distribution of a general consumer awareness handout about family history of high blood pressure. The distribution at these churches also had the advantage of using an established, respected support system.

## Methods

**Figure:**
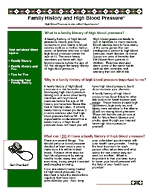
Download the *Family History and High Blood Pressure* handout (PDF–95K)

MDCH and UM-CGPH collaborated to develop the content of the *Family History and High Blood Pressure* handout. The content was initially developed by the UM-CGPH staff and later refined by MDCH staff over a six-month period. A worksheet for individuals to identify their own personal family history is a key part of the material. Readability levels using Flesch–Kincaid were conducted, and the material was judged to be at an approximate eighth-grade level. Drafts of the handout were circulated to ten MDCH staff members to test understanding of the directions and provide general feedback on clarity. After modifications, the material was printed in a colorful handout.

The introductory page of the handout includes three questions on knowledge of, attitude toward, and preventive actions for a family history of high blood pressure. The handout includes a worksheet that encourages collection of the family history of high blood pressure, heart disease, and stroke, including age of onset and age and cause of death. The consumer is encouraged to start with first-degree relatives (parents and siblings) and then to extend to second-degree relatives (grandparents and aunts/uncles). This approach of adding affected relatives and age of onset provides a simple overview for risk assessment, which can be used by families and their health care providers. The worksheet also emphasizes the importance of sharing the collected information with medical providers and family and discussing screening and healthy lifestyles with offspring. The last page highlights that high blood pressure is a chronic condition requiring evaluation and treatment.

The proposal for this project was presented to the *Blood Pressure Sunday* planning group. The planning group includes four parish nurses, two American Heart Association staff members, two professional volunteers, and one MDCH cardiovascular health nurse consultant.  Members agreed that the material could be introduced to parish nurses and other health coordinators at the one-day training session. During this training, a 15-minute overview of *the Family History and High Blood Pressure* pilot was given, and a sample of the handout was distributed to all participants. At the conclusion of the training, 12 churches in Detroit agreed to pilot the *Family History and High Blood Pressure* handout. A follow-up contact was made to review the pilot and estimate the number of handouts for each church. *Family History and High Blood Pressure* handouts were sent to each church.

## Consequences

Approximately 500 copies of the *Family History and High Blood Pressure* handout were distributed. The pilot was discussed at a follow-up meeting with the parish nurses; more than half of the participating parish nurses were in attendance. Written feedback was obtained prior to the follow-up meeting. In addition, there was a discussion about successes and challenges in the pilot. Overall, the participating parish nurses expressed similar experiences and concerns about the utility of information collected. The possibility that the consumer may not remember to share the family history information at his or her next medical appointment was discussed. Also, the important question of whether this information would be a motivator to change behavior was raised. The parish nurses thought that consumers understood the material, comprehended its importance, and took interest in the topic. Some consumers reportedly commented to the nurses that they had never thought about how high blood pressure in their family related to their own health.

Overall, this pilot had several strengths: handouts were distributed to a population at high risk for high blood pressure; a collaborative effort was achieved between an academic center and state health department chronic disease and genomics programs; genomics information was successfully integrated into an existing program; the ability to reach churches in a predominantly black community was demonstrated; the consumers reported interest in the subject matter; the difficult task of attaining an appropriate literacy level of the material was advanced; and a worksheet format for a family history tool was developed. The worksheet format is simple, inexpensive, and easily integrated into other chronic disease materials.

Limitations of this pilot include a lack of field testing for consumers' opinion about the readability of the handout. We would like to have evaluated the audience's understanding of the information and whether the proper messages were conveyed. Also, while the MDCH Cardiovascular Health and Genomics Programs received indirect feedback about the tool from the church nurses, the programs and the nurses did not systematically collect information from the consumers on the usefulness of the handout and the worksheet.

## Interpretation

The MDCH Genomics Work Group and two additional CDC-funded state genomics programs reviewed this pilot project. Possible suggestions for the future include changing the handout in the following ways:

- Modifying the format (e.g., using bullet points to reduce verbiage, separating the educational component from the family history tool for clarity and utility);
- Revising current content (e.g., simplifying instructions for family history worksheet, further lowering the literacy level); and
- Adding more information (e.g., additional chronic diseases, incorporating culturally appropriate messages and images).

The development and use of a general consumer awareness handout is an iterative process. Therefore, the next steps for the program include further enhancements of the handout, field testing of the handout and worksheet, and collaboration with other possible channels of dissemination such as physicians and local health departments.

This pilot of a general consumer awareness handout on family history and high blood pressure demonstrated the challenges of developing material on this complicated topic. One suggestion is to implement this kind of activity at a place where participants can read the material, complete a feedback form, and return for discussion. Possible settings for this scenario include waiting rooms at physicians' offices prior to routine physicals or waiting rooms at local health departments prior to health screenings and/or immunizations. This context would provide a direct and immediate communication with a medical provider to highlight the family history and prevention messages. Future plans include distributing this handout at a science museum in Detroit, targeting a younger audience in a nonreligious setting. 

The circumstances of this pilot limited the ability to collect feedback information on behavior and attitude change. It was hoped that this type of feedback would be gained from the parish nurses. Because of time constraints, however, collection of feedback was not feasible. Future initiatives should include collection of follow-up information. For instance, a focus group with participants could strengthen the content of the handout and provide feedback on methods to collect follow-up information.

In summary, this pilot project was a worthwhile activity that will be adapted for use in other settings. Additionally, the collaborative process of developing and distributing the handout is an example of how to integrate genomics into existing resources within chronic disease programs in other state health departments.

## Figures and Tables

**Table:** Possible Risk Factors for High Blood Pressure in Detroit^a^

**Suspected Higher Risk Groups for High Blood Pressure^a^**	**Detroit Population^b^**
Family history of high blood pressure	Unknown
African American ancestry	Majority African American
Over 65 years	Not relevant^c^
Lower socioeconomic status	One of lowest household incomes in large U.S. cities
Overweight or obese	High proportion of obese or overweight respondents
Sedentary lifestyle	Highest proportion of no physical activity in leisure time
Excess intake of dietary sodium and/or insufficient intake of potassium	Unknown
Excess alcohol consumption	Not relevant^c^

a Source: National High Blood Pressure Education Program (3).

b Sources: *Detroit in focus: a profile from Census 2000* (13) and Bureau of Epidemiology, Michigan Department of Community Health (7,14).

c Michigan BRFSS data do not appear to support this risk factor for the Detroit population.
