# Supplementary material for: Blood Pressure Sunday: Introducing Genomics to the Community Through Family History
Source: Prev Chronic Dis. 2005 Mar 15;2(2):A23. (PMC1327717)
Supplement: Supplementary file 1 [file 04_0134_01.pdf]

Family History and High Blood Pressure*

High Blood Pressure is also called ‘Hypertension’

Find out about these topics:

- Family History
- Family History and You
- Tips for You
- Knowing Your Family History

What is a family history of high blood pressure?

A **family history of high blood pressure** means you have someone in your family (a blood relative such as a mother, father, sister, or brother) who has or had high blood pressure before the age of 60. The more family members you have with high blood pressure before the age of 60, the stronger the family history of high blood pressure.

High blood pressure tends to run in families for many reasons. Blood relatives tend to have many of the same *genes* that can predispose a person to high blood pressure, heart disease, or stroke. *Genes* are units of heredity that are passed from parents to children. Relatives may also share some of the same habits such as diet, exercise, and smoking that can affect risk.

Why is a family history of high blood pressure important to me?

A family history of high blood pressure is a risk factor for you developing high blood pressure. Having one or more close family members with high blood pressure before the age of 60 means you have **two times the risk** of having it also. A **strong** family history means you have 3 or more relatives who had high blood pressure before 60. It is **important to understand** that a family history of high blood pressure does not mean you will have high blood pressure, but it does increase your chances.

A family history of high blood pressure has been linked to other risk factors for heart disease and stroke. These factors include high cholesterol, high body fat, and being more sensitive to the effects of salt on raising blood pressure. These risk factors can put you at risk for future heart disease and stroke, even though you may not have high blood pressure yourself.

What can I DO if I have a family history of high blood pressure?

There are several things. You should get your blood pressure checked at least once a year to make sure it is within normal levels. Reduce other risks for high blood pressure by eating healthy foods, using less salt, exercising, losing weight if needed and stopping smoking.

If you are already being treated for high blood pressure, it is important to take the medications regularly that have been prescribed for you. Also, keep your scheduled appointments with your health care provider. Finding the best treatment for each person often takes time and what works for one person may not work for another. **What is important** is that you keep trying to lower your blood pressure with the help of your health care provider!

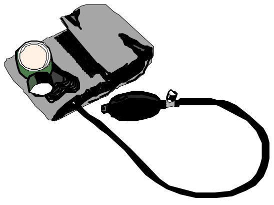

Get Checked!

How do I learn about MY family history of high blood pressure, heart disease or stroke?

You can fill out **My Family Health History Worksheet** on the next page. This worksheet will help you to identify members of your family and health information on each of them.

How to fill out the Family Health History Worksheet

Step 1: List your family members. Start with those who are closest to you in relation, such as your mother, father, brothers, and sisters. (If you want, you can then list other relatives that are more distant in relation, such as grandparents, aunts and uncles.)

Step 2: Next to each family member, check (✓), if they have high blood pressure, heart disease, or stroke.

Step 3: If you know the age they got these diseases, write that down. If they died, write down when they died and what caused them to die. See the example below.

	High Blood Pressure	Heart Disease	Stroke
Mother Susan	✓ Got at age 34		✓ Died at 60 of a stroke
Father Bill	✓ Got at 58	✓ Got at 59	
Brother (s) None			
Sister (s) None			
Total # of (✓)	2	1	1

DRAFT

If you have **more than one close relative that developed these diseases before the age of 60**, then you are at an increased risk of these diseases. You can stop here or keep going by adding other members of your family.

SHARE !

Once you have completed your family history worksheet, keep it and make updates from time to time. You can show your doctor so that he or she can better understand your risk for certain diseases. Discuss it with your children so they understand their risks and the need for getting their blood pressure checked often and practicing healthy habits.

MY FAMILY HEALTH HISTORY WORKSHEET

Immediate Family:

	**High Blood Pressure**	**Heart Disease**	**Stroke**
Mother			
Father			
Brother(s)<br>__________________<br>__________________<br>__________________<br>__________________			
Sister(s)<br>__________________<br>__________________<br>__________________<br>__________________			
**Total # of (✓)**			

Other Relatives:

(Mother's side)<br>Grandfather<br>Grandmother<br>Aunt(s)<br>Uncle(s)			
(Father's side)<br>Grandfather<br>Grandmother<br>Aunt(s)<br>Uncle(s)			
**Total # of (✓)**			

DRAFT

If you or a family member has high blood pressure, here are some tips to know.

Goal Blood Pressure levels:

New guidelines suggest ideal blood pressure is below 120/80.

If you have diabetes, heart or kidney disease, it is ideal to have your blood pressure below 140/90 and even lower.

Uncontrolled high blood pressure can result in a stroke, kidney disease, heart attack or other health conditions.

High blood pressure is almost always a **life-long condition** requiring ongoing medical evaluation and treatment. It is important to stay under a physician's care and follow the treatment regimen prescribed to keep the blood pressure in recommended levels.

Treatment of high blood pressure usually includes some dietary recommendations and medication.

The preferred **diet** for controlling high blood pressure is the DASH eating plan. This plan emphasizes eating higher amounts of fruits and vegetables, lower sodium, lower fat, whole grain and low fat diary foods. This diet is rich in magnesium, potassium, calcium and fiber with adequate protein. For more information about this diet, you can obtain a copy from www.nhlbi.nih.gov or check with your physician.

Many different types of **medications** are now available to treat high blood pressure. They work in many different ways, so some people will need to take two or more to bring the blood pressure down to controlled levels. Because the medications work differently, it may take trials of several types to see what works best for you. Do not be discouraged because this is not unusual. Keep working with your health care provider to find the medication that lowers your blood pressure and does not cause any negative effects.

Controlling high blood pressure is very important. Since high blood pressure usually has no symptoms, the only way to know if it is high is **to have it checked**. **Keep records** of your blood pressure readings and the treatment programs you are on. Being an **active participant in your care** will increase the success of life-long control.

For more information check www.nhlbi.nih.gov or www.americanheart.org
